# Prevalence and Risk Factors Associated With Carpal Tunnel Syndrome Among Sudanese Females: A Cross-Sectional Study

**DOI:** 10.7759/cureus.72943

**Published:** 2024-11-03

**Authors:** Gaffar Abbas, Mohamed B Ahmed, Fatima S Almohannadi, Khaled E Elzawawi, Ahmed B Ahmed, Abeer Alsherawi

**Affiliations:** 1 Department of Medical Education, Hamad General Hospital, Hamad Medical Corporation, Doha, QAT; 2 College of Medicine, Qatar University (QU) Health, Qatar University, Doha, QAT; 3 Department of Plastic Surgery, Hamad General Hospital, Hamad Medical Corporation, Doha, QAT; 4 Physical Therapy, Faculty of Physical Therapy, Heliopolis University, Cairo, EGY

**Keywords:** cts, females, obstetrics and gynecology, pregnancy, risk factors

## Abstract

Background: Carpal tunnel syndrome (CTS) is the most common entrapment neuropathy resulting from compression of the median nerve. This condition is more common in females than in males. The earlier the diagnosis, the better the prognosis and treatment outcomes.

Material and methods: This is a cross-sectional hospital-based study conducted at Soba Teaching Hospital in obstetrics and gynecology outpatient clinic in Khartoum in the period from February 2022 to March 2022. A simple random sampling was applied, and an interview was conducted using a four-part questionnaire. The first part was based on socio-demographic data, the second was lifestyle, and the third and fourth were based on the Boston Carpal Tunnel Questionnaire (BCTQ). The data were analyzed using the statistical package for social sciences (SPSS) software, version 23 (IBM Corp., Armonk, NY) to find the correlation between the various variables.

Results: A total of 113 women participated in the study (response rate=100%). The prevalence of CTS was found to be 5%. No significant association was found between lifestyle and medical history, e.g., obesity and oral contraceptives and CTS were p=0.167, 0.841 respectively. Conversely, there was a significant association between age and residency, with p=0.005 and 0.049, respectively.

Conclusions: The prevalence of CTS is increasing in Sudan. Therefore, it is essential to thoroughly assess patients for any symptoms related to hand pain by asking detailed questions during consultations. In addition, further studies covering more states are needed to better understand the regional variation in CTS prevalence.

## Introduction

Carpal tunnel syndrome (CTS) is recognized as being the most common entrapment neuropathy with an incidence of 1 to 5% in the general population [1]. Entrapment neuropathy happens when a nerve gets compressed, usually by other structures around it or by fluids through edema [2]. The median nerve can become compressed as it passes through the two heads of the pronator teres muscle which causes pronator teres syndrome [3]. Moreover, in the case of CTS, the median nerve gets compressed as it passes through the carpal tunnel at the level of the wrist which is the more common level of median nerve entrapment [4,5]. CTS can be unilateral, affecting only one hand, or bilateral, depending on multiple factors.

The median nerve innervates the muscles of the thenar compartments of the palm, flexor pollicis longus, abductor pollicis brevis, and opponens pollicis. The palmar cutaneous branch of the median nerve innervates the skin over the thenar eminences and lateral three-and-a-half digits on the palmar aspect of the hand. Over the dorsum of the hand, the median nerve provides sensation to the skin over the distal two-and-a-half digits till the proximal interphalangeal (PIP) joints and to the skin over the thumb distally till the interphalangeal (IP) joint [2,4]. Thus, the compression of the median nerve in CTS produces symptoms mainly in the palm, thumb, index, middle finger, and radial side of the ring finger of the affected hand. The symptoms include pain, burning sensation, tingling sensation, numbness, and muscle weakness in the digits of the affected hand. Symptoms vary in severity across patients, and they are classified into mild, moderate, and severe [6].

Risk Factors for CTS include female gender, pregnancy, obesity, repetitive wrist activity, genetic heredity, and inflammatory conditions such as rheumatoid inflammation, hypothyroidism, hypertension, and diabetes mellitus [1,6,7]. Female predominance of the condition has been noted with a female-to-male ratio of 3:1 [1]. This predominance is hypothesized to be caused by females having a relatively narrower carpal tunnel compared to their male counterparts, which in turn increases the chance of median nerve entrapment over time [8]. Another theory of why there is a female predominance stems from an occupational point of view. For instance, it theorizes that females can get CTS in part because their occupations or tasks require repetitive and extensive wrist use, which over time leads to median nerve entrapment [9]. The mechanism by which pregnancy causes CTS is similar to that of edema, as hormonal shifts in the female body lead to fluid retention combined with musculoskeletal changes, causing median nerve compression by the increased fluids [10].

CTS prevalence seems to be increasing in the general population over time. The clinical implications of CTS extend beyond just the physical symptoms; the condition can significantly reduce an individual's quality of life. Symptoms such as pain and muscle weakness can impact performance in both professional and personal aspects of life. In severe cases, chronic pain and severe muscle weakness can lead to psychological impacts as well, including depression and decreased self-esteem [11]. Finally, if left untreated, CTS may result in irreversible median nerve damage, leading to permanent disability. Although several studies investigated the prevalence and risk factors of CTS among females worldwide, there still seems to be a notable lack of evidence among females in Africa. This is especially important due to the cultural impact in this region, as females usually are the backbone of the family and the community with a wide variety of critical tasks at hand. The presence of a limited healthcare system in the region, combined with the lack of evidence of CTS prevalence and distinct regional risk factors, further complicates this dilemma. Thus, this study aims to examine the prevalence of CTS and investigate the potential risk factors associated with CTS development among females in a tertiary teaching hospital's Obstetrics and Gynecology Outpatient Clinic in Sudan.

## Materials and methods

This is a cross-sectional hospital-based study conducted in the Obstetrics and Gynecology Outpatient Clinic, Soba Teaching Hospital, Khartoum, Sudan in the period from the 1st of February 2022 to the 31st of March 2022. The facility was established in 1975 as a tertiary hospital; later, it was converted into a teaching hospital affiliated with Khartoum University. Soba Teaching Hospital is located in Khartoum state in the margin of the capital, which makes the hospital a destination for urban and rural residents with different tribal backgrounds and ethnic differences, giving a good reflection of the general population of Sudan. Moreover, the hospital is famous for its high-quality facilities and competent doctors, and patients with a variety of socio-economic statuses can be seen in the clinic. The sample targeted all the Sudanese females attending the clinic; hence, females above the age of 20 years were included. The hospital is located in an area with diverse nationalities; hence, non-Sudanese women were excluded from the study. Also, women below the age of 20 years were excluded. Assuming a prevalence rate of 0.08 at 95% CI with a margin of error of 5%, the required sample size was estimated to be 113. Data accumulation in this study is based on a non-probability sampling approach. Data collection was performed through interviews and specially designed versions of the Boston Carpal Tunnel Questionnaire (BCTQ) [12]. The questionnaire is divided into two major sections: symptom severity scale and functional status scale. Scores were calculated for each section, and any deviation from the normal scale for any section was considered abnormal. All analyses were performed using SPSS software, version 23 (IBM Corp., Armonk, NY). In this model, conditions related to CTS are considered dependent variables. Age, lifestyle factors (alcohol consumption or smoking), general medical conditions including diabetes, thyroid diseases, autoimmune disorders, and reproductive health-related factors related to pregnancy and oral contraceptive drugs are all independent variables. Ethical approval was obtained from the Department of Community Medicine, Faculty of Medicine, University of Khartoum(approval number COMMED 2022-93-13). Informed written consent was obtained from all participants, and an assurance of confidentiality was given in this research.

## Results

The demographic analysis showed that most of the female respondents were relatively young, and a significant number (43%) fell within the age bracket of 20-29 years. In Addition, most of the participants (91%) were married, which emphasizes the dominance of married females in this study. Moreover, nearly half of the participants (42%) fall within the middle-income class. An overview of the educational achievement of the participants reveals that 43% have acquired a university degree or higher qualification, representing a relatively high level of education in the population sample under study (Table 1).

**Table 1:** Socio-demographic characteristics of the study population

Variable		N	%
Age (years)	20-29	49	43%
	30-39	40	35%
	40-49	10	9%
	50-59	9	8%
	>60	5	4%
Marital status	Single	5	4%
	Married	103	91%
	Divorce	1	1%
	Widow	4	4%
Family monthly income	Less than 50000	34	30%
	50000-100000	48	42%
	More than 100000	31	27%
Education	Illiterate	7	6%
	Primary school	11	10%
	Secondary school	46	41%
	University degree and above	49	43%
Residence	Urban	70	62%
	Rural	43	38%
Occupation	Housewife	97	86%
	Student	7	6%
	Employee	9	8%
BMI	Underweight	7	6%
	Normal	35	31%
	Overweight	37	33%
	Obese	21	19%
	Extremely Obese	13	12%
	Total	113	100%

The distribution of participants based on the residential location was that 62% reside in urban areas. This shows that the majority of participants in this research study are from the city background. Also, it has been recorded from the study that the highest percentage, 86 %, of participants are housewives. This occupational predominance might have the potential to influence the study's outcome since the lifestyle and activities associated with housework may be related to the variables being investigated. Furthermore, the distribution observed shows that most participants fall out of the normal weight range, as a fair portion falls within the categories of overweight 33% and obese 19% (Table 1).

The analysis of the relation between various lifestyle and health factors and the diagnosis of CTS, by using the Chi-square test and its p-value, indicated that none of the variables examined (smoking, alcohol consumption, diabetes, thyroid disease, overweight, fractures, contraceptive use, pregnancy, menstrual disorders, or autoimmune diseases) were significantly associated with CTS in this study (Table 2).

**Table 2:** Different variables and their relation to CTS with its chi square and p-value CTS: Carpal Tunnel Syndrome.

		Are you diagnosed with Carpal Tunnel Syndrome?						Chi-Square P-value
		Yes		No		Total		
		N	%	N	%	N	%	
Do you smoke cigarettes or shisha?	Yes	0	0%	2	100%	2	100%	0.735
	No	6	5%	105	95%	111	100%	
Do you drink alcohol?	Yes	0	0%	1	100%	1	100%	0.812
	No	6	5%	106	95%	112	100%	
Do you have diabetes?	Yes	0	0%	8	100%	8	100%	0.487
	No	6	6%	99	94%	105	100%	
Do you have any thyroid diseases?	Yes	1	11%	8	89%	9	100%	0.418
	No	5	5%	99	95%	104	100%	
Do you suffer from obesity?	Yes	4	9%	41	91%	45	100%	0.167
	No	2	3%	66	97%	68	100%	
Have you ever had a fracture in your upper limbs?	Yes	1	11%	8	89%	9	100%	0.418
	No	5	5%	99	95%	104	100%	
Have you ever used or still using oral contraceptives?	Yes	3	6%	49	94%	52	100%	0.841
	No	3	5%	58	95%	61	100%	
Have you ever experienced pregnancy?	Yes	6	6%	97	94%	103	100%	0.433
	No	0	0%	10	100%	10	100%	
Are you pregnant now?	Yes	2	4%	55	96%	57	100%	0.389
	No	4	7%	52	93%	56	100%	
Have you ever experienced menstrual period disorders?	Yes	1	6%	15	94%	16	100%	0.856
	No	5	5%	92	95%	97	100%	
Do you have any autoimmune disease?	Yes	0	0%	2	100%	2	100%	0.735
	No	6	5%	105	95%	111	100%	

The study indicates a significant association between people aged between 40-49 years and CTS diagnosis with a p-value of 0.005. Another significant association between urban area residents and CTS diagnosis with a p-value of 0.049. Other groups showed no significant association (Table 3).

**Table 3:** Age and residence association with being diagnosed with CTS as p-value originated by using chi-square

		Are you diagnosed with Carpal Tunnel Syndrome?						Chi-Square P-value
		Yes		No		Total		
		N	%	N	%	N	%	
Age	20-29	1	2%	48	98%	49	100%	0.005
	30-39	1	3%	39	98%	40	100%	
	40-49	3	30%	7	70%	10	100%	
	50-59	1	11%	8	89%	9	100%	
	>60	0	0%	5	100%	5	100%	
Residence	Urban	6	9%	64	91%	70	100%	0.049
	Rural	0	0%	43	100%	43	100%	

**Figure 1:**
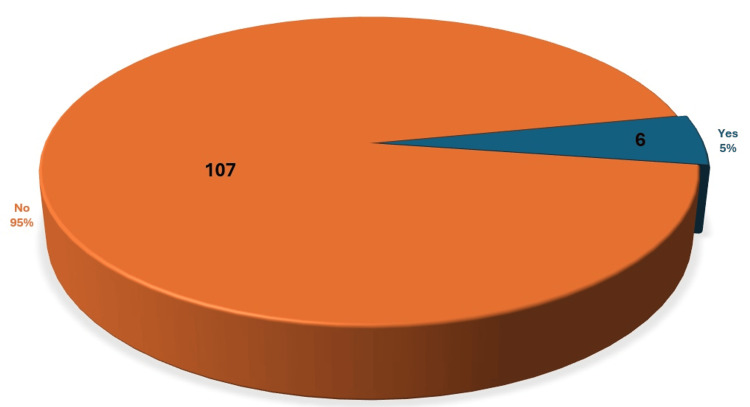
Percentage of CTS among participants as 5% have CTS and 95% are normal CTS: Carpel Tunnel Syndrome

**Figure 2:**
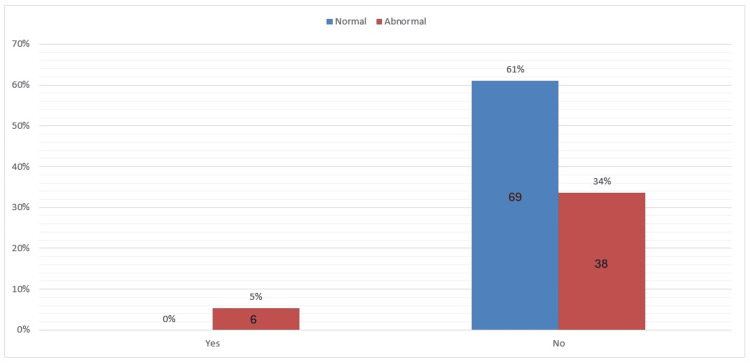
The symptoms severity scale (BCTQ) divided into normal and abnormal outcome and the corresponding diagnostic result Diagnostic result: yes= diagnosed, no= not diagnosed BCTQ: Boston Carpal Tunnel Questionnaire.

**Figure 3:**
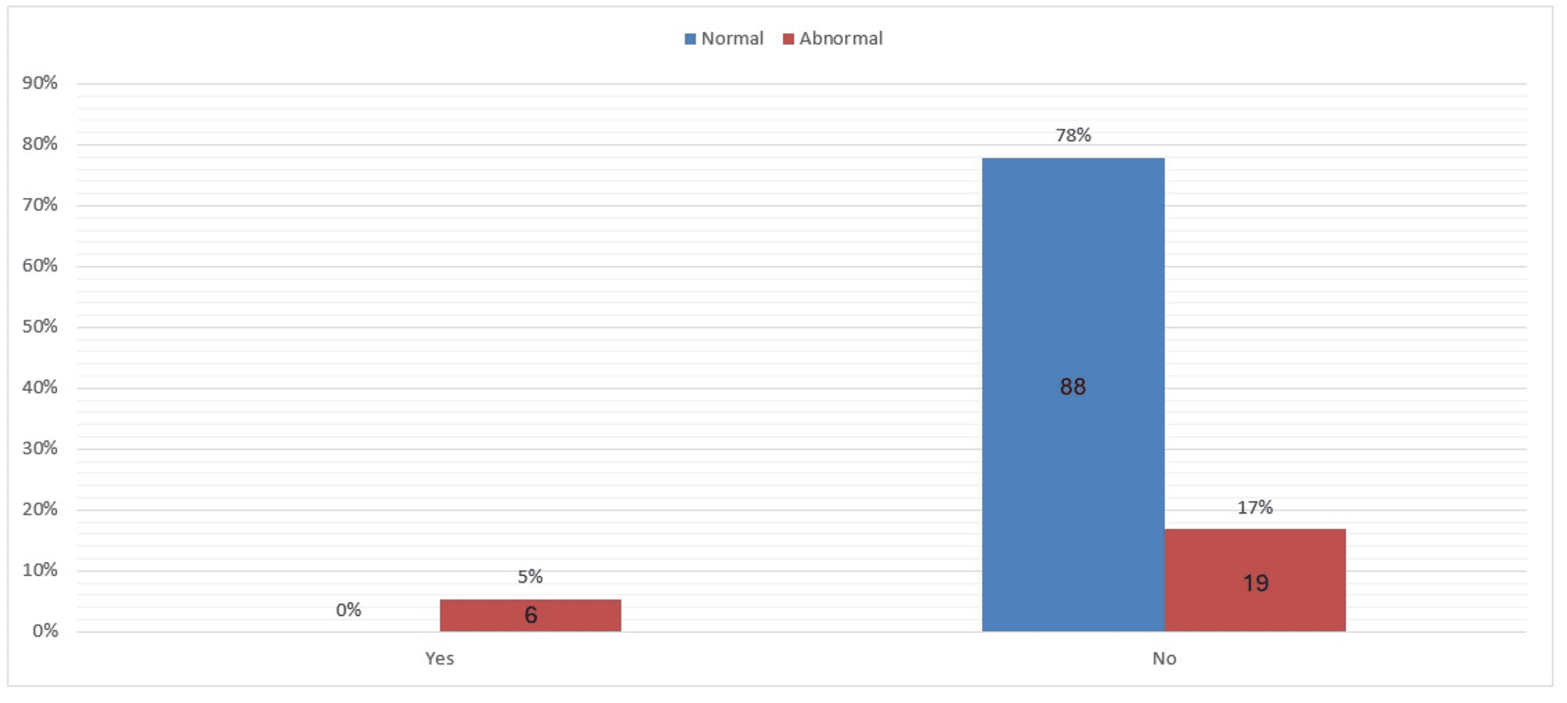
The functional status scale (BCTQ) divided into normal and abnormal outcome and the corresponding diagnostic result Diagnostic result: yes= diagnosed, no= not diagnosed BCTQ: Boston Carpal Tunnel Questionnaire.

Only 5% of participants were diagnosed with CTS, and the remaining 95% were not clinically diagnosed. However, a surprising finding was that 34% of participants had abnormal symptoms severity scale outcome as well as 17% of the participants had abnormal functional deficit scale outcome without being diagnosed with CTS. These results indicate that those participants have higher chances of having CTS yet undiagnosed (Figures 1-3).

## Discussion

The prevalence rate of CTS among Sudanese females attending the Obstetrics and Gynecology Outpatient Clinic at Soba Teaching Hospital was found to be 5%, which is similar to the rate observed in the general population [13]. This picture aligns with expected ranges from other regions, reinforcing the understanding that CTS is a common condition. In addition, the prevalence of CTS consistently shows significant gender differences, with women being three times more likely to develop the condition than men. One paper suggests that this finding is due to anatomical differences, as females tend to have relatively narrower carpal tunnels, which can contribute to nerve entrapment [14]. Moreover, as presented in this investigation, age was one of the significant risk factors among the studied population, in particular, with participants between the ages of 40 and 49 years who recorded the highest prevalence of CTS, p=0.005. According to previous studies, age has been considered one of the critical factors in the development of CTS [15]. The reason behind this observation is that age always correlates with physiological changes, such as degeneration of soft tissues and reduced elasticity of nerves, leading to median nerve compression. These age-related changes make this age group more susceptible to developing CTS. From the results, urban residence also showed a significant association with CTS at p=0.049; this may be explained by the lifestyle factors associated with urban residence, which include repetition in using the hands at work. More et al. attributed this to urbanization, which increases exposure to jobs requiring continuous wrist action that predisposes one to CTS after some time [16]. Interestingly, the study found no significant association between obesity, the use of oral contraceptives, and other lifestyle factors with CTS. This is contrary to what other studies propose that factors such as obesity, oral contraceptives, and lifestyle are connected to the increased risk for CTS [17]. This may be because of a discrepancy in sample size, genetic predispositions, or regional lifestyle variations.

The study showed that even though 34% had abnormal symptom severity scale results and were not diagnosed with CTS. The same findings were noted in the functional status scale results, with 17% being undiagnosed with abnormal results. This highlights the issue of underdiagnosis, particularly in regions with limited access to healthcare. According to one nested case-control study, people in rural areas where health services are not available might not visit hospitals even when they experience mild or moderate symptoms. Underdiagnosis also occurs in urban areas, despite better access to healthcare facilities, primarily due to a lack of routine screening [18]. Another unexpected finding is that as many as 86% of the participants in the current study were homemakers. Certain occupational factors, such as repetitive hand movements involved in cleaning, cooking, and childcare, may predispose a person to CTS. These tasks are not considered professional work; however, they involve continuous wrist activity similar to that found in manual work in professional settings. The results from the studies conducted in non-professional settings, like housework, indicate an increased hazard for CTS associated with repetitive use of the hands [19]. This finding points out that homemakers may be equally exposed to the same occupational hazards as people undergoing formal employment with repetition of hand movements. These findings carry significant implications for Sudanese women, as routine screening in obstetrics and gynecology clinics will increase awareness. Since urban residency is a risk factor, special attention should be given to urban-dwelling women, whose daily lives may be challenging due to repetitive hand use in the workplace [18]. The risk factor profile highlights the necessity for public health interventions aimed at reducing the risk of CTS and ensuring early diagnosis among urban women [17]. The results also identify that CTS has a high vulnerability to women aged 40-49 years. The age group should be a priority in health programs that deal with the prevention and management of CTS since physiological changes linked to aging might be destructive enough to increase the chances of median nerve compression. Early diagnosis in these populations can significantly reduce symptom development and enhance overall quality of life [14]. Another area of concern is the high percentage of homemakers in this study, most of whom engage in repetitive hand movements during domestic tasks. Since housework is often an overlooked risk factor, it presents significant occupational hazards for the development of CTS. One paper argues that interventions should be implemented in health care to raise awareness among homemakers regarding the possible danger of repeated hand movements and promote preventive measures, such as ergonomic practices and regular medical check-ups [20]. This could reduce the prevalence of CTS and generally improve the health status of Sudanese women by addressing occupational risks distinctive to this population.

Several limitations could be stated about the current study. First, this research was done in one hospital in Khartoum; thus, generalization to all Sudanese women is not possible. Also, data was self-reported, and that can introduce bias or inaccuracies because participants may have underreported or misunderstood their symptoms. This, together with the relatively small number of respondents, 113 in all, further limits the ability to make broader conclusions on the prevalence of CTS and risk factors within the wider population. Therefore, further studies on CTS among Sudanese women need to be extended over different regions of Sudan as that will give a broader perspective on the prevalence and risk factors of CTS across the country. In addition, healthcare professionals, especially those in outpatient clinics, should be trained in the identification and diagnosis of CTS to ascertain the early stages of asymptomatic cases of CTS and prevent further damage. This might enable early detection and also ensure that women receive proper care before the situation becomes intolerable.

## Conclusions

In conclusion, the prevalence of CTS among Sudanese females in obstetrics and gynecology outpatient clinics in Soba Teaching Hospital is 5%. Age and residency were found to have a significant association with CTS diagnosis. Moreover, key outcomes identified in our paper, like the high likelihood of undiagnosed cases presenting with positive signs and symptoms, as well as the consequences associated with delayed diagnosis, require further research. Additional studies might help in shedding light on factors that may help in the early diagnosis and management of these cases to achieve better outcomes.
